# Partial Anterior Cruciate Ligament (ACL) Tears in Adolescents: Are We Overtreating Them or Underestimating Who Should Not Wait?

**DOI:** 10.7759/cureus.114522

**Published:** 2026-08-14

**Authors:** Javier Gonzalez Almonacid

**Affiliations:** 1 Orthopedics, San Borja Arriarán Clinical Hospital, Santiago, CHL; 2 Orthopedics, German Clinic of Santiago, Santiago, CHL

**Keywords:** adolescent, anterior cruciate ligament, nonoperative treatment, partial acl tear, pediatric sport medicine

## Abstract

Partial anterior cruciate ligament (ACL) tears in adolescents remain clinically challenging because of their heterogeneous presentation and uncertain functional prognosis. Although traditionally considered less severe than complete ruptures, these injuries encompass a highly heterogeneous spectrum, ranging from functionally competent ligaments that maintain clinical stability and permit return to activity after rehabilitation to mechanically insufficient knees that rapidly progress to instability and secondary intra-articular damage. Accordingly, a major clinical challenge may lie not simply in choosing between surgery and nonoperative treatment, but in accurately identifying which adolescents are unlikely to succeed with conservative management. Available observational cohorts report progression to ACL insufficiency or delayed reconstruction in approximately 25%-39% of patients initially managed nonoperatively, although validated predictors of failure remain unavailable. The term partial ACL tear describes ligament morphology but does not necessarily reflect ligament function, because residual fibers may preserve sufficient biomechanical stability despite structural disruption, whereas some morphologically partial tears remain mechanically insufficient. Formal clinical prediction tools have not been adequately evaluated or externally validated, and existing studies have assessed individual clinical and imaging variables in small, heterogeneous cohorts with inconsistent findings. Therefore, treatment decisions should rely on comprehensive functional assessment, skeletal maturity, sporting demands, and the risk of secondary injury rather than MRI appearance alone. Reframing the debate in this way may improve patient selection and optimize treatment decisions for this complex injury.

## Editorial

Anterior cruciate ligament (ACL) injuries in children and adolescents are increasingly encountered, with a 20-year epidemiological analysis reporting an average annual incidence increase of 2.3% and peak rates during the high-school years [1]. The optimal management of partial ACL tears remains controversial, with treatment options ranging from structured rehabilitation to selective bundle augmentation or complete ACL reconstruction [2]. Traditionally, the debate has been framed as a balance between avoiding unnecessary surgery and preventing the consequences of delayed reconstruction. However, the central problem may not be whether we operate too frequently or wait too long, but whether the diagnosis of a partial tear provides enough information to guide either decision.

The term partial ACL tear implies an injury of lesser severity, yet it encompasses a remarkably heterogeneous group of patients. Some adolescents retain a functionally competent ligament and successfully return to sport after structured rehabilitation, whereas others develop instability, secondary meniscal injury, and ultimately require reconstruction. Although MRI can demonstrate structural abnormalities and associated injuries, its reported diagnostic accuracy for partial ACL tears is only 25%-53%; specific MRI signs have reported sensitivities of 45%-82% and specificities of 53%-97%. Diagnostic performance varies according to technical image quality and protocol, reader expertise, and the diagnostic criteria applied, and MRI morphology alone cannot determine whether the residual ligament remains functionally competent [2]. Although direct visualization and probing during arthroscopy remain the diagnostic reference standard, arthroscopy should not be performed solely to confirm a suspected partial tear and is generally reserved for patients already undergoing operative management [2,3]. The same diagnostic label may therefore include patients with fundamentally different prognoses.

This heterogeneity also explains why the literature has struggled to establish a universally accepted definition of a partial ACL tear. As summarized in Table 1, this variability is reflected in a narrative review and observational cohort studies rather than in a single standardized definition: some studies rely on MRI findings and clinical stability, whereas others incorporate instrumented laxity, the estimated proportion of injured fibers, bundle involvement, or arthroscopic confirmation [2-5]. Consequently, treatment decisions based primarily on imaging or an arbitrary percentage of intact fibers may lead to errors in both directions.

**Table 1:** Comparison of definitions, diagnostic criteria, and prognostic findings across representative studies of partial ACL tears ACL: anterior cruciate ligament; MRI: magnetic resonance imaging; OR: odds ratio.

Study	Design/population	Definition and diagnostic criteria	Main prognostic findings	Clinical implication
Stone et al. [2]	Narrative review incorporating adult and adolescent literature.	Partial tears are classified as functional or nonfunctional. A functional ligament allows confident return to sport with minimal or no laxity after rehabilitation; a nonfunctional ligament produces symptomatic instability or objective laxity. Diagnosis integrates history, physical examination, instrumented laxity, and MRI, with arthroscopy remaining the reference standard.	Reviewed series reported progression to complete rupture in 14%–56% of young active patients. Younger age, pivoting contact sports, symptomatic instability, and failure to return confidently to sport were considered relevant.	Morphological continuity does not necessarily indicate functional competence. Management should prioritize knee function, sporting demands, and response to rehabilitation rather than MRI appearance alone.
Kocher et al. [3]	Prospective cohort of 45 patients aged ≤17 years with arthroscopically confirmed partial tears, initially treated nonoperatively; mean follow-up 6.1 years.	Acute hemarthrosis, MRI signal abnormality, grade A or B Lachman and pivot shift under anesthesia, and arthroscopic confirmation. Tears were classified as ≤50% or >50% and as predominantly anteromedial or posterolateral.	Fourteen patients (31%) required reconstruction. Involvement of >50% and posterolateral tears were independent predictors; older skeletal age and grade B pivot shift were also associated with failure in selected analyses.	Nonoperative treatment may succeed in carefully selected stable adolescents, but tear extent and bundle involvement identify clinically different prognostic groups.
Fayard et al. [4]	Level III retrospective analysis of prospectively collected data from 41 active patients aged <30 years; mean age 20.6 years, range 14–28; mean follow-up 43 months.	Delayed but firm Lachman endpoint, side-to-side laxity ≤4 mm measured with the Rolimeter, negative pivot shift, and MRI showing a partial rather than complete tear.	Sixteen patients (39%) progressed to complete rupture. Age ≤20 years (OR 5.19) and participation in pivoting contact sports (OR 6.29) increased the risk; 50% of progressing patients developed meniscal injury.	Clinically stable patients may still progress. The findings support caution in young pivoting athletes, although extrapolation to adolescents is limited by the inclusion of adults aged 21–28 years.
Hannon et al. [5]	Level III retrospective pediatric cohort with follow-up survey of 84 patients aged 8–18 years; mean age 13.8 years; median follow-up 6.3 years.	Clinic-based diagnosis using traumatic history, physical examination, and MRI. Patients with findings suggesting complete rupture, including a positive pivot shift or Lachman with a soft or absent endpoint, were excluded.	Twenty-one patients (25%) progressed to ACL insufficiency, at a median of 11.4 months after return to sport. No routinely assessed demographic, clinical, MRI, rehabilitation, or bracing variable reliably predicted progression.	Partial tears diagnosed through routine clinical data carry a meaningful risk of failure, but currently available variables do not provide validated individual risk stratification.

As summarized in Table 1, this conclusion is based primarily on a narrative review and observational cohort studies rather than systematic reviews or formal expert consensus. Collectively, these studies suggest that tear extent and bundle involvement may influence prognosis, but morphology alone does not reliably determine treatment success; clinical stability, symptoms, sporting demands, and response to rehabilitation are also required to assess whether the residual ligament remains functionally competent [2-5]. These variables are derived primarily from observational cohort studies and narrative review rather than from high-quality prospective validation or formal expert consensus. Accordingly, clinical stability, pivot shift, patient age, skeletal maturity, sporting demands, and confidence during return to pivoting activities should currently be regarded as components of clinical judgment rather than validated predictive variables, and should be integrated with MRI findings rather than interpreted in isolation [2-5]. A partially intact ligament is not necessarily a functional ligament.

The consequences of misclassification may be substantial. In the classic pediatric series by Kocher et al., nearly one-third of children and adolescents initially managed without reconstruction ultimately required delayed surgery. Fourteen of 45 patients (31%) ultimately underwent ACL reconstruction. Reconstruction was required in 53% of tears involving more than 50% of the ligament versus 18% of smaller tears, in 50% of predominantly posterolateral tears versus 16% of anteromedial tears, and in 100% of patients with a grade B pivot shift versus 23% with a grade A pivot shift; however, only tear extent greater than 50% and posterolateral bundle involvement remained independent predictors in multivariate analysis [3]. Fayard et al. reported progression to complete rupture in 39% of a mixed cohort of adolescents and young adults aged 14 to 28 years. Although this overall rate cannot be directly extrapolated to a pediatric population, patients aged 20 years or younger had a significantly greater risk of progression [odds ratio (OR) 5.19], and secondary meniscal injuries were present in 50% of those who progressed [4]. More recently, Hannon et al. found that 21 of 84 pediatric patients (25%) initially treated nonoperatively developed ACL insufficiency over a median follow-up of 6.3 years [interquartile range (IQR), 3.5-9.7 years]. Among patients with complete return-to-sport data, progression occurred a median of 11.4 months (IQR, 5.5-28.6 months) after return to sport, while routinely assessed clinical, imaging, and treatment variables did not reliably predict failure [5].

These findings should not be interpreted as evidence that every adolescent with a partial ACL tear requires reconstruction. Carefully selected patients with stable knees, minimal laxity, absent or low-grade pivot shift, and favorable functional characteristics may still be successfully managed with structured rehabilitation, particularly when substantial growth remains [3]. Nevertheless, conservative treatment should be understood as a structured, monitored therapeutic trial rather than passive observation. In the absence of a validated protocol, a pragmatic approach consists of approximately three to four months of progressive rehabilitation, with reassessment at approximately six weeks and again before return to pivoting sport. Monitoring should combine recurrent giving-way episodes or effusion, Lachman and pivot-shift findings or instrumented laxity, strength and neuromuscular recovery, sport-specific functional testing, and serial patient-reported outcomes. Published cohorts have used the International Knee Documentation Committee (IKDC) subjective score, Tegner Activity Scale, and anterior cruciate ligament - return to sport after injury avale (ACL-RSI) to assess knee function, activity level, and psychological readiness, although no validated cutoff on any of these measures defines treatment failure in adolescents with partial ACL tears. Recurrent instability, worsening objective laxity, persistent inability to meet functional return-to-sport criteria, deteriorating patient-reported function, or progression to a complete tear or secondary intra-articular injury should prompt reconsideration of the initial strategy [3-5].

Rather than continuing to debate surgery and rehabilitation as opposing approaches, future research should focus on identifying which partial tears remain functionally competent and which have already become mechanically insufficient. Priority should be given to prospective multicenter cohorts using standardized diagnostic definitions, serial clinical and functional assessments, instrumented laxity testing, and patient-reported outcomes. Biomechanical studies could clarify how residual fiber continuity relates to anterior and rotational stability, while externally validated prediction models integrating age, skeletal maturity, sporting demands, examination findings, and imaging characteristics may improve individualized risk stratification. Until reliable prediction models are available, treatment decisions should rely less on MRI morphology alone and more on repeated functional assessment, skeletal maturity, sporting demands, and the potential consequences of recurrent instability. A partial ACL tear is a morphological description, not a treatment algorithm. A pragmatic, nonvalidated decision-making framework is to first determine whether the knee is functionally stable, then assess the response to a structured rehabilitation trial, and finally consider patient age, skeletal maturity, pivoting-sport demands, tear pattern, and the potential consequences of recurrent instability. Continued nonoperative care may be appropriate when clinical stability and functional progression are maintained, whereas recurrent giving-way, worsening objective laxity, inability to meet return-to-sport criteria, or secondary intra-articular injury should prompt early consideration of reconstruction. The greatest challenge is therefore not choosing between surgery and rehabilitation in every adolescent, but recognizing which patients may safely undergo a structured trial of nonoperative care and which appear to warrant earlier reconstruction. Even after careful clinical, functional, and imaging assessment, substantial uncertainty remains; decisions should therefore be individualized through shared decision-making with adolescents and their families, considering skeletal maturity, sporting goals, tolerance for risk, and the potential consequences of recurrent instability.
